# Social Media for Global Neurosurgery. Benefits and limitations of a groundbreaking approach to communication and education

**DOI:** 10.1016/j.bas.2023.101728

**Published:** 2023-03-11

**Authors:** Alfredo Conti, Marcello Magnani, Matteo Zoli, Ralf A. Kockro, Constantin Tuleasca, Simone Peschillo, Giuseppe Emmanuele Umana, Seow Wan Tew, George Jallo, Kanwaljeet Garg, Robert F. Spetzler, Jesus Lafuente, Bipin Chaurasia

**Affiliations:** a Department of Neurosurgery, IRCCS Istituto delle Scienze Neurologiche di Bologna Alma Mater Studiorum Università di Bologna, Via Altura 3, 40123, Bologna, Italy; b Centre for Microneurosurgery, Hirslanden Hospital, Zurich, Switzerland; c Lausanne University Hospital (CHUV), Neurosurgery Service and Gamma Knife Center, Lausanne, Switzerland; d University of Lausanne (UNIL), Faculty of Biology and Medicine (FBM), Switzerland; e Ecole Polytechnique Fédérale de Lausanne (EPFL, LTS-5), Lausanne, Switzerland; f Unicamillus—Saint Camillus International University of Health Sciences, Rome, Italy; g Trauma Centre, Gamma Knife Centre, Department of Neurosurgery, Cannizzaro Hospital, Catania, Italy; h Department of Neurosurgery, National Neuroscience Institute, Singapore; i Institute of Brain protection sciences, Johns Hopkins All Children's Hospital, St. Petersburg, Florida, USA; j Department of Neurosurgery and Gamma Knife, All India Institute of Medical Sciences, New Delhi, India; k Barrow Neurological Institute, Phoenix, AZ, USA; l Department of Neurosurgery, Hospital Universitari Sagrat Cor, Barcelona, Spain; m Department of Neurosurgery, Neurosurgery Clinic, Birgunj, Nepal

**Keywords:** Global neurosurgery, Social media, Facebook, Education, Neurosurgery, Limitations

## Abstract

**Introduction:**

Social media have become ubiquitous and their role in medicine is quickly growing. They provide an open platform by which members share educational material, clinical experiences, and collaborate with educational equity.

**Research question:**

To characterize the role of social media in neurosurgery, we analyzed metrics of the largest neurosurgical group (Neurosurgery Cocktail), collected relevant data about activities, impact and risks of this groundbreaking technology.

**Material and methods:**

We extracted Facebook metrics from 60-day time sample, including users demographics and other platform-specific values such as active members and number of posts within 60 days. A quality assessment of the posted material (clinical case reports and second opinions) was obtained establishing four main quality-criteria: privacy violation; quality of imaging; clinical and follow up data.

**Results:**

By December 2022, the group included 29.524 members (79.8% male), most (29%) between 35 and 44 years of age. Over 100 countries were represented. A total of 787 posts were published in 60 days with an average of 12.7 per day. In 173 clinical cases presented through the platform, some issue with privacy was recorded in 50.9%. The imaging was considered insufficient in 39.3%, clinical data in 53.8%; follow up data were missing in 60.7%.

**Discussion and conclusion:**

The study provided a quantitative evaluation of impact, flaws and limitations of social medial for healthcare. Flaws were mostly data breach and insufficient quality of case reports. There are actions to correct these flaws that can be easily taken to provide a greater credibility and efficacy to the system.

## Highlights

• Quantitative evaluation of the pros and cons of social media use in neurosurgery.
• Analysis of the most successful neurosurgical group (Neurosurgery Cocktail).
• Over 100 collaborative published studies trough the platform.
• Main flaws represented by data breach and insufficient quality of case reports.
• Light corrections needed to provide greater credibility and efficacy to the system.

## 1 Introduction

Social media are virtual spaces within which individual or institutional, public or semi-public, or anonymous profiles may electronically communicate and exchange information though web-based platforms (Grajales et al., 2014). They have become ubiquitous in everyday life, and their role in medicine is quickly growing. Social media indeed provide an open platform by which members can share educational material, compare clinical experiences, and collaborate with educational equity (Nicolosi et al., 2018; Servadei et al., 2018; Budohoski et al., 2018). Within these virtual spaces, communities with interests covering nearly all possible fields spontaneously emerged. One of these groups was established in 2016 and named “Neurosurgery cocktail” (NC). It was developed by a group of independent neurosurgeons to facilitate the announcement of activities and share clinical experiences among neurosurgeons worldwide. The group turned out to be astonishingly successful, quickly raising to more than 35.000 neurosurgeons across the globe on multiple platforms, but predominantly active on Facebook and Twitter.

The benefit for healthcare of such large, instantly and continuously accessible forums are indeed limitless. To mention a few, they represent the only independent and unfiltered window on the global practice of neurosurgery. Secondly, neurosurgeons can now interact with others, sharing opinions in real time. Thirdly, spontaneous aggregation of neurosurgeons from distant areas of the world and with different neurosurgical and economic background can rapidly produce scientific collaboration and practice changes. On the other hand, there are potential dangers especially related to uncontrolled and horizontal diffusion of materials and opinions, poor methodological approach to decisions, lack of follow up data. The size of these flaws still needs to be investigated.

To characterize the role of social media within the field of neurosurgery, we analyzed the features of the largest neurosurgical group reporting on demographics and web traffic and collected relevant data to identify the activities, impact and risks of this groundbreaking approach to neurosurgeons’ interaction.

## 2 Methods

### 2.1 Metrics of the group

We extracted Facebook metrics parameters through the platform's analytics tools from 23^rd^ October 2022 to 22^nd^ December 2022. The metrics reviewed were the number of platform users/followers, user demographic information (age, sex, country of origin), and several platform-specific values such as active members and number of posts within 60 days.

### 2.2 Quantitative and qualitative analysis of published material

A detailed analysis of the *Neurosurgery cocktail*'s content was performed together with those of other neurosurgery groups on Facebook, taking into account a 60-day timespan ranging from October 23rd^,^ 2022 to December 22nd^,^ 2022. The first step consisted in the definition of an organized workflow by three main analytical phases:

• phase 1: categorization of published content into three main categories
• phase 2: evaluation of number of global and per post interactions, intended as the number of likes and comments
• phase 3: quality assessment of the posted content

Particularly, during phase 1, three main categories were identified: a) *clinical cases*, regarding either clinical case reports (fully presented case management and results) and second opinions (cases for which an advice about the management of complex cases was asked to the community); b) *knowledge sharing*, i.e. shared articles, congress reports, congress invitations, webinars and didactical images, videos and links; c) *miscellaneous*, a section regarding random images, videos or unclassifiable elements.

For the phase 3, a quality assessment of the posted material, exclusively regarding clinical case reports and second opinions, was obtained by establishing four main quality-criteria: *privacy violation*, according to the categories of identifiable information under the Health Insurance Portability and Accountability Act (Fargen et al., 2021); *low-quality imaging*, including inappropriate imaging modality for the case, insufficient imaging data (i.e. planes, slices, etc.), and low-quality images; *insufficient clinical data*, as the lack or scarceness of clinical information; *lack of outcome or follow-up data*, evaluating if after the presentation and discussion of a case, further information regarding the evolution or conclusion of the matter were provided.

After the collection of all the data, statistical inferences were performed, in particular:

- Quantitative analysis: total published posts, number of published posts per category and average of post published per day.
- Interaction analysis: total likes and comments number, identification of the preferential category (based on total interaction) in a one-month duration sample and the evaluation of interactions' distribution among *clinical case* and *knowledge sharing* categories in a ten-days duration sample.
- Qualitative analysis: assessment of *clinical cases*-category quality level based on the four above-mentioned criteria being either fulfilled (N) or infringed (Y). Analysis was performed independently by two reviewers. In cases of disagreement, an second unblinded re-evaluation of score assigned to published material was performed to find a final agreement.
- Qualitative analysys of comments: comments added to posts were classified as “relevant” or “not relevant”. A comment was deemed “relevant” whether following criteria were fulfilled: a) the comment was coherent with the subject treated in a given post; b) the comment was written using a clear and comprehensible medical vocabulary; c) the comment was either an answer to a clinical question or a question regarding a posted clinical case.

The comment was not considered relevant if it was just limited to an emoticon, GIF or sticker, congratulations, acknowledgements, or greetings. Any kind of sponsored or commercial interaction with posted was deemed not relevant.

### 2.3 Collaborative products

We retrieved all scientific collaborative products produced by direct interactions among members and through the social media platforms, including: published papers, surveys, webinars, congresses organizations, and other relevant initiatives.

Article published were classified as follows: a) *Basic research*: papers concerning basic neurological investigations; b) *General neurosurgery*: papers regarding vascular, oncological, traumatological and spinal surgery; c) *Functional neurosurgery*; d) *Global Neurosurgery*: papers regarding the use of social media, the perspectives of the discipline in a given country or other more general and less clinical issues.

*Not classified*: not-available papers (removed from the Facebook page or not found in Pubmed or other browsers) or duplicates.

## 3 Results

### 3.1 Metrics of the group

**Fig. 1:**
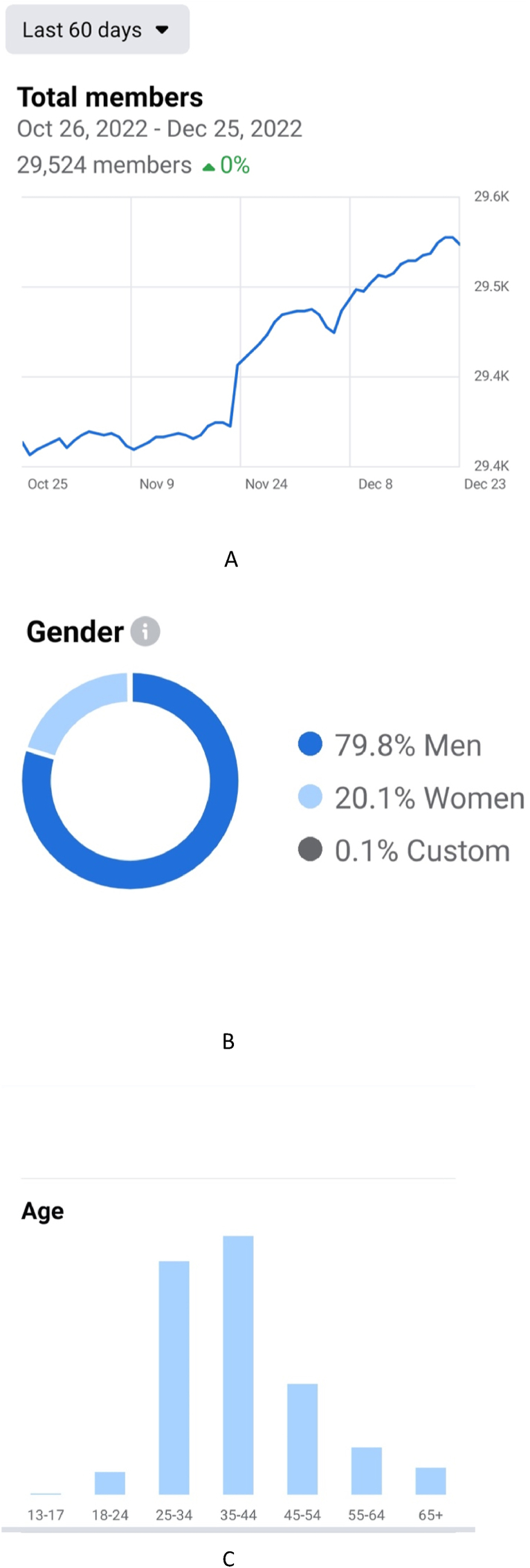
Facebook metrics graphs. A) By December 2022, the *neurosurgery cocktail* group had a total of 29,524 members; B) Gender distribution of group members; C) Age categories of member. The largest age group (29%) included members 35–44 years old.

The NC Facebook group was created on 16 April 2016. By December 2022, the group had a total of 29,524 members (Fig. 1A). Female members accounted for 20.2%, mirroring the current global gender disparity (Garozzo et al., 2022). (Fig. 1B). Before admission, each profile was screened by the group managers in order to admit only medical students, neurosurgical residents or board certified neurosurgeons. The largest age group was between 35 and 44 years of age. (29%), that of junior specialists followed by that between 25 and 34, namely that of residents (27%) (Fig. 1C).

**Fig. 2:**
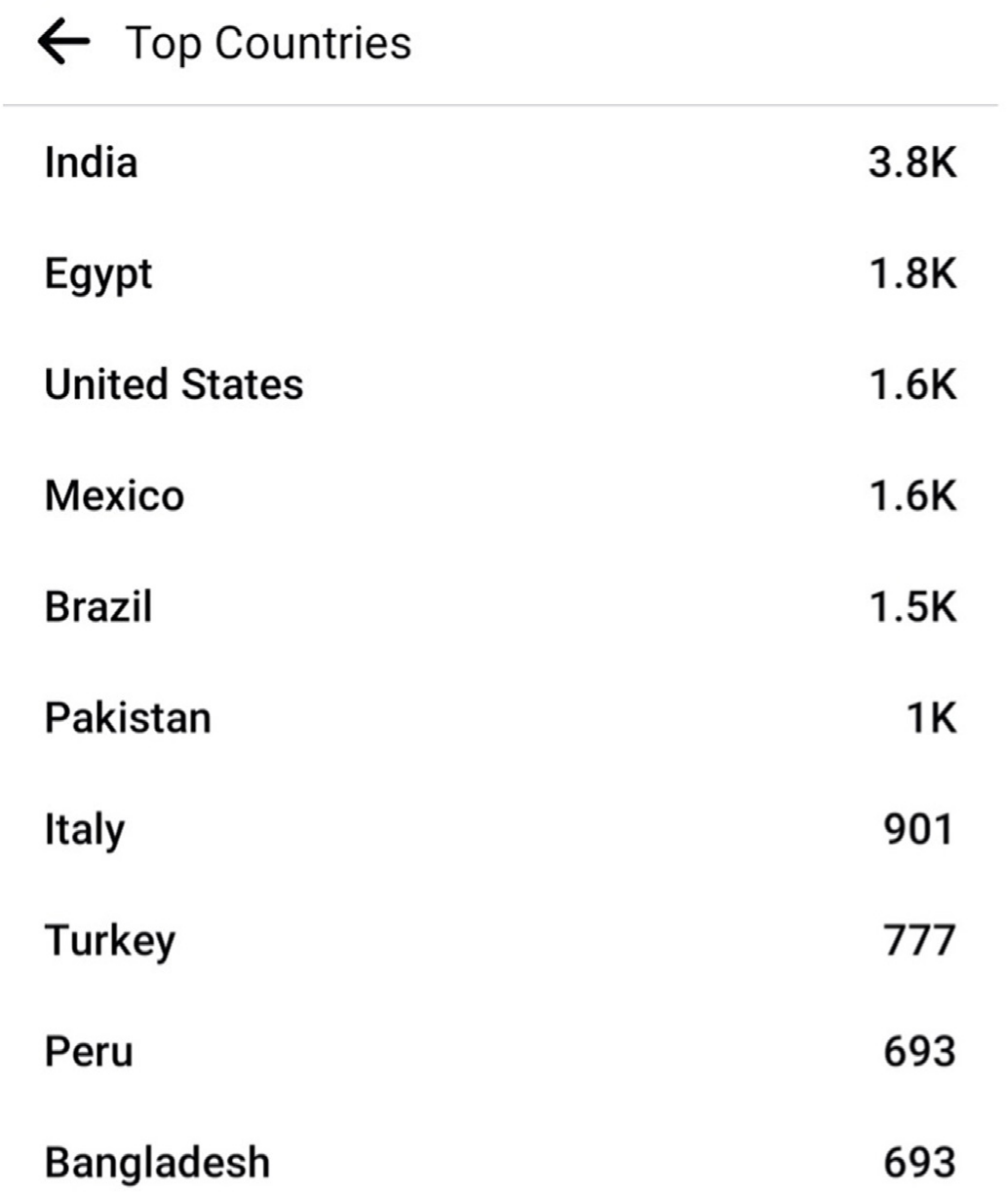
Over 100 countries were represented in the group; India was most prominently represented with 3,800 members (12.9%), followed by Egypt with 1800 members (6.1%) and USA with 1600 members (5.4%).

Over 100 countries were represented; India was most prominently represented with 3,800 members (12.9%), followed by Egypt with 1800 members (6.1%) and USA with 1600 members (5.4%) (Fig. 2).

### 3.2 Quantitative analysis of published material

A total of 787 posts were published from October 22nd, 2022 to December 22nd, 2022 with an average of 12.7 posts per day (Fig. 3A). We detailed the post typologies (Fig. 3B):

- Clinical cases: 77 were 2nd opinions and 96 were clinical case reports. Total: 173 (22%).
- Knowledge sharing: 57 papers, 235 congress reports and invitations, webinars reports, 269 didactical images, videos or shared links. Total: 561 (71.3%).
- Miscellaneous: 53 posts (6.7%).

**Fig. 3A:**
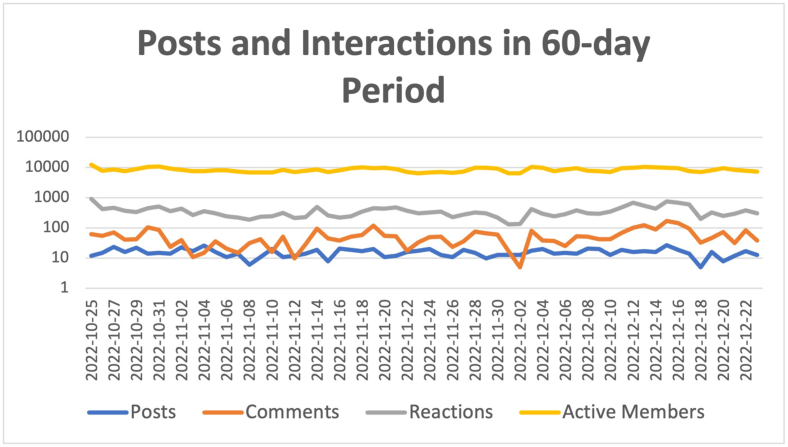
Number of active members, number of posts, reactions and comments in a 60-day study period.

**Fig. 3B:**
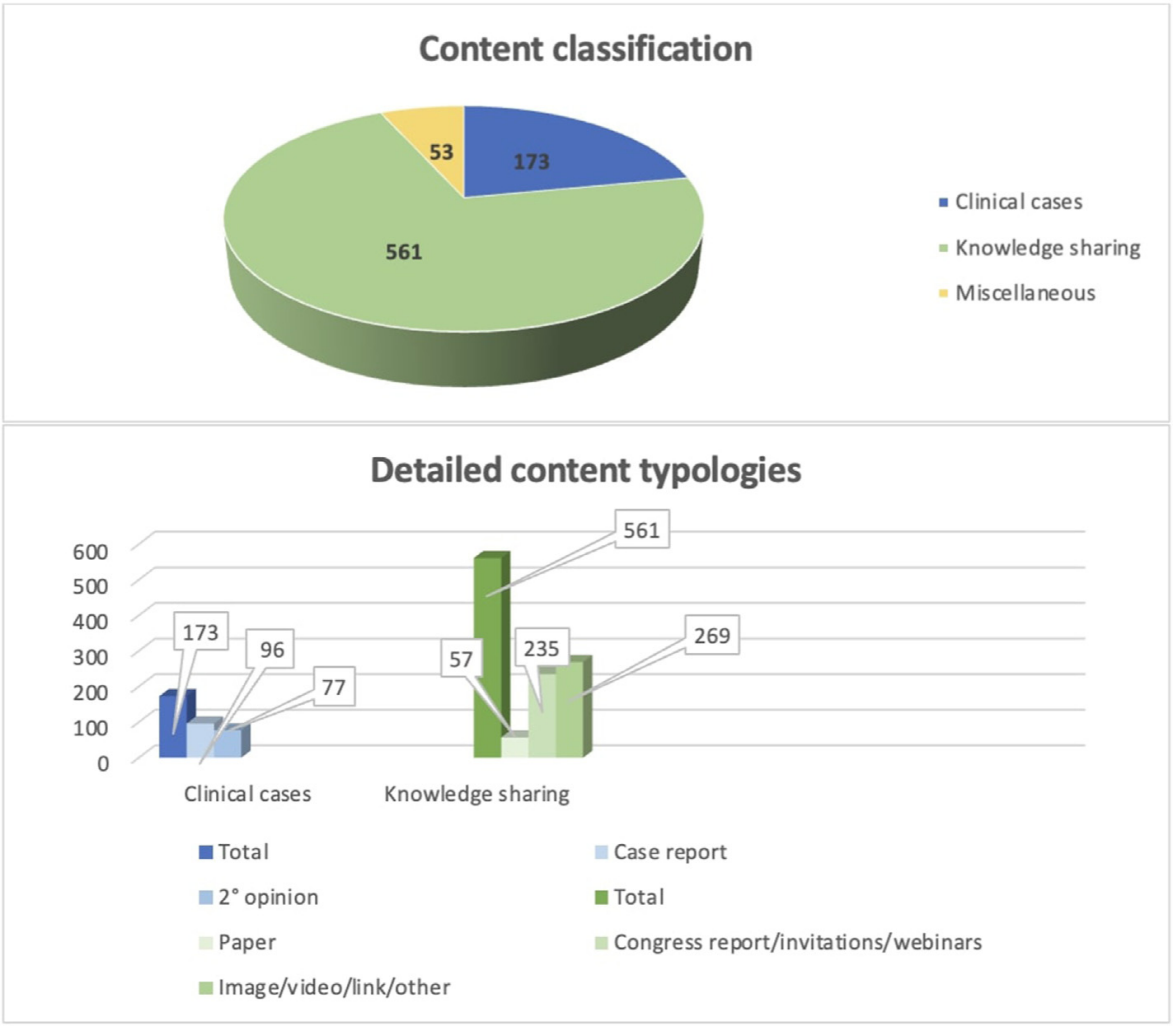
Content of posts as categorized in three main categories (upper): clinical cases, knowledge sharing, miscellaneous. Detailed content typologies (lower): incuding second opinions, case reports and research papers, images, videos, links and other research materials, congress reports, invitations, webinars with relative numbers as collected in a 60-days interval.

#### 3.2.1 Interaction analysis

A total of 15,566 likes and 2,919 comments were submitted in the analyzed 60-day period. A 30-days sample (from November 24th^,^ 2022 to December 22nd^,^ 2022) was collected and analyzed in order to identify the category receiving the highest degree of attention and interaction. The *clinical cases*-category carried the highest number of reactions (likes and comments) for 21 out of the total 30 days.

We then analyzed the distribution and type of interactions for posts falling in the category “case report” from December 13th to December 22nd. A total of 3051 likes and 844 comments were retrieved (Fig. 4A). *Clinical cases*-category accounted for a total of 2279 (74.7%) likes and 844 (88.7%) comments. Then, we also analyzed the proportion accounting for the subcategory *case reports* versus *second opinions*. The results showed that case reports accounted for a total of 1834 out of 2279 (80.5%) likes and 462 out of 749 (61.7%) comments; second opinions accounted for a total of 445 out of 2279 (19.5%) likes and 287 out of 749 (38.3%) comments (Fig. 4B).

**Fig. 4A:**
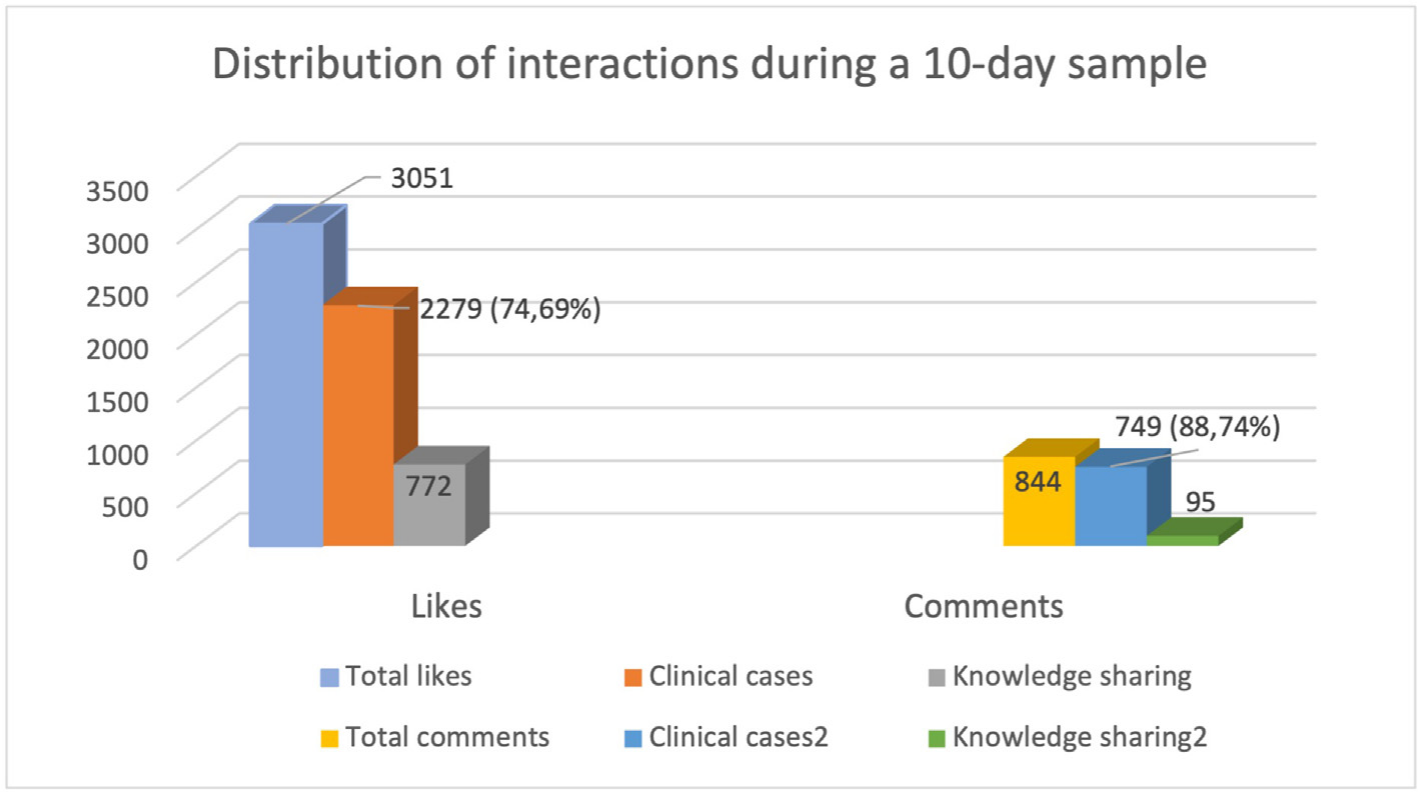
Distribution of interactions (likes and comments) during a 10-day sample.

**Fig. 4B:**
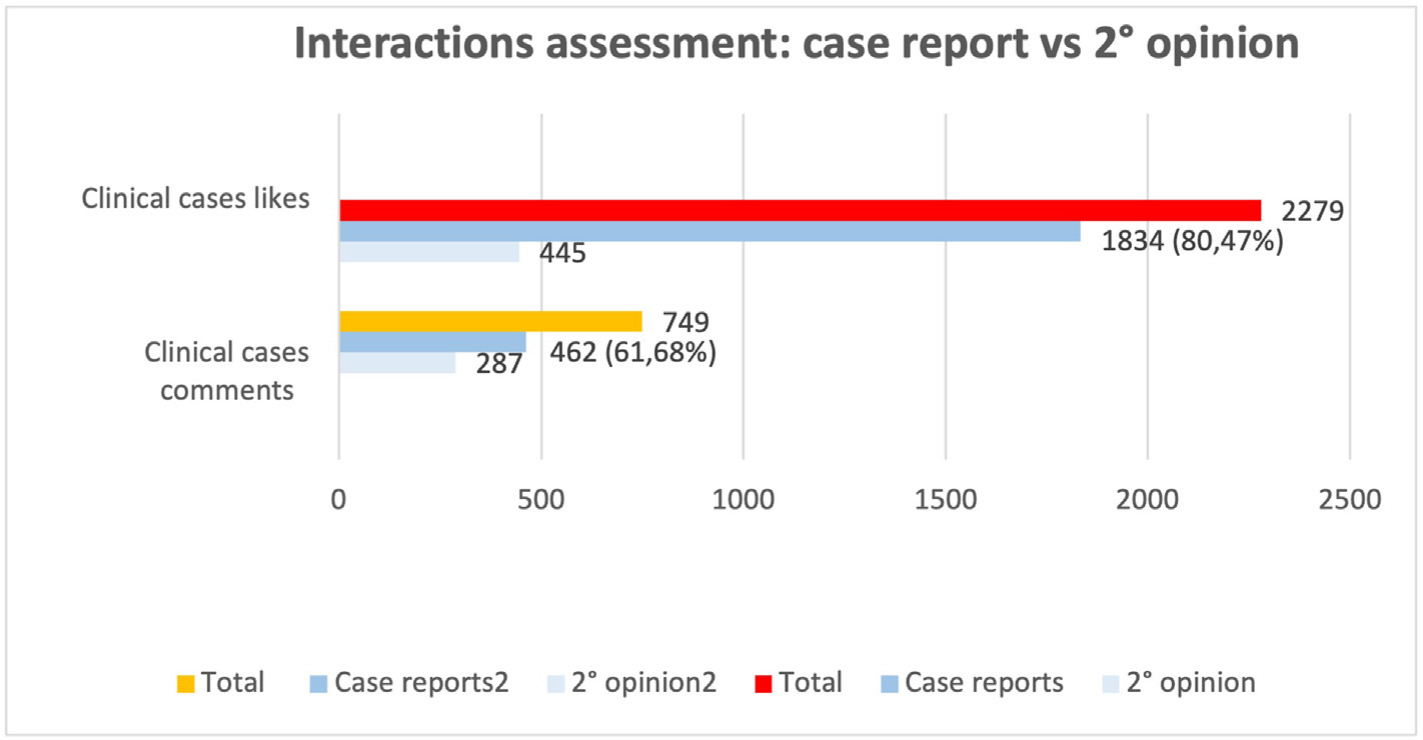
Distribution of interactions (likes and comments) during a 10-day sample for two categories of posts (clinical cases and second opinions).

#### 3.2.2 Qualitative analysis

**Fig. 5:**
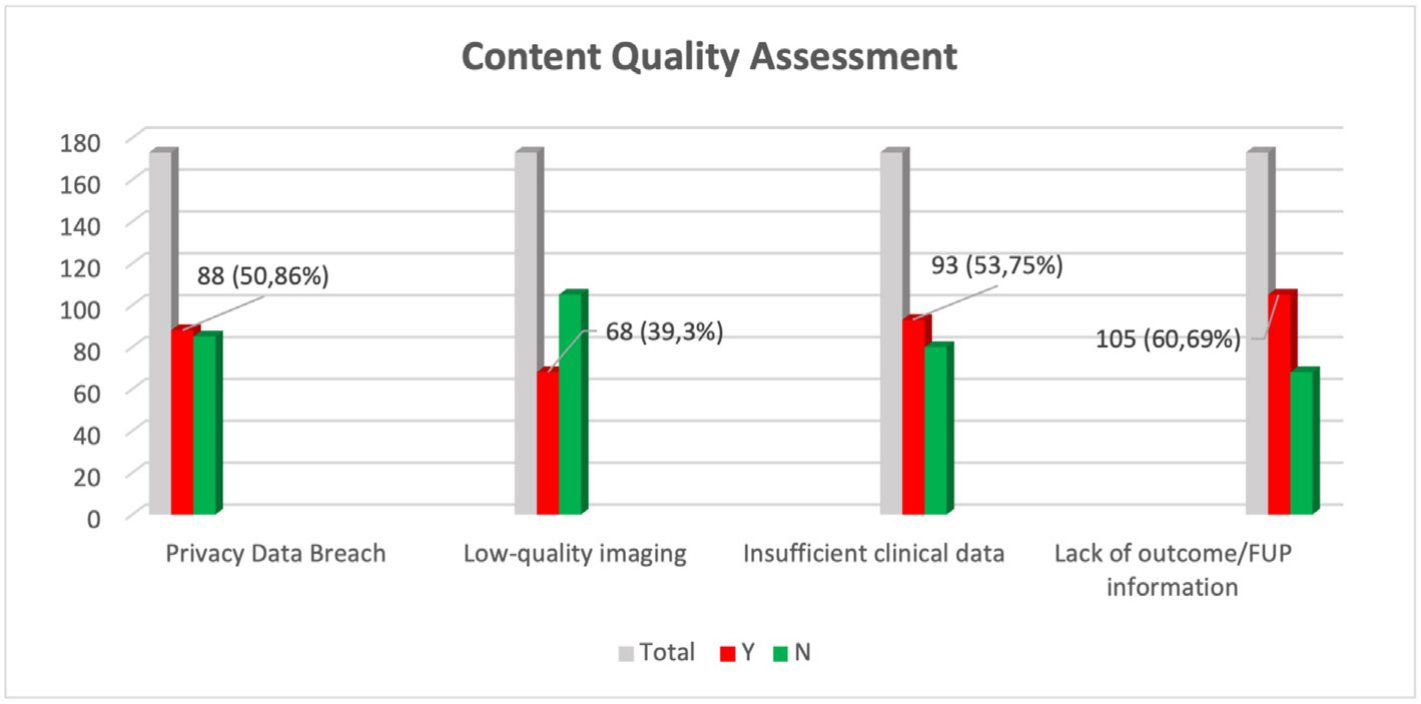
Bar graph showing the content quality assessment. Total number of cases (grey bar); flawed cases unflawed cases (green bar) were reorted with relative percentages. (For interpretation of the references to colour in this figure legend, the reader is referred to the Web version of this article.)

Finally, an analysis was performed in order to evaluate the quality of data reporting. The quality control was performed exclusively on the *clinical cases*-category, as this particular category was naturally prone to these specific risks (Fig. 5). Inter-rater agreement was found in 159/173 posts, with 14 (8.1%) requiring an open revision between two reviewers.

- Privacy infringement: in 88 out of 173 posts (50.9%), violation of privacy data protection occurred. The most frequent error regarded the publishing of personal health information (PHI) as dates (date of birth, date of the examinations) or inappropriate terminology as “two days ago” “last month” which can make posted content susceptible to PHI data leaks. In some cases, the patient was clearly identifiable.
- Low-quality imaging: in 68 out of 173 posts (39.3%), either image quality was insufficient or imaging data were missing.
- Insufficient clinical data: in 93 out of 173 posts (53.8%), crucial clinical data were briefly and insufficiently reported.
- Lack of outcome or follow-up (FUP) data: in 105 (60.7%) out of 173 posts, outcome or follow up data were missing or insufficient to allow an evaluation of the case management.
- Qualitative analysis of interactions: According to the criteria selected for qualitative analysis of interactions with published materials, of 2919 comments/interactions, 1937 (66.4%) were deemed relevant and 982 (33.6%) not relevant.

### 3.3 Comparison with other Facebook groups

We analyzed other major neurosurgical groups in the same social media platform and including >10k followers. Three groups were retrieved and analyzed:

I. *Neurosurgery*: 24.995 members
II. *Neurosurgery platform*: 11.930 members
III. *Neurosurgery news and cases*: 10.544

The analytical process was performed according to the same criteria used for the analysis of “Neurosurgery Cocktail”and results summarized in Table 1.

**Table 1:** Quantitative and qualitative metrics of published material in major neurosurgical groups in Facebook (including >10k followers).

GENERAL INFORMATION		CONTENT QUANTATIVE ANALYSIS		CONTENT CLASSIFICATION			INTERACTIONS		QUALITATIVE ANALYSIS			
GROUP NAME	MEMBERS	TOTAL POSTS	DAILY SHARING ACTIVITY	CLINICAL CASES	KNOWLEDGE SHARING	MISCELLANEOUS	LIKES	COMMENTS	PRIVACY VIOLATION	LOW-QUALITY IMAGING	INSUFFICIENT CLINICAL DATA	LACK OF OUTCOME & FUP DATA
*Neurosurgery cocktail*	29,524	787	12.7	173	561	53	15.566	2.919	50.86%	39.3%	53.7%	60.7%
*Neurosurgery*	24,995	127	2.04	5	87	35	415	37	0%	20%	40%	40%
*Neurosurgery platform*	11,930	203	3.3	6	154	43	141	22	33.3%	33.3%	33.3%	33.3%
*Neurosurgery news and cases*	10,544	118	1.9	3	57	58	43	19	33.33%	66.7%	33.3%	66.7%

### 3.4 Collaborative products

The NC group promoted more than 50 surveys, 15 of which have since been retrieved as published papers in PubMed (Chaurasia et al., 2022; Fontanella et al., 2020; Deora et al., 2020, 2022; Tandon et al., 2021; Robertson et al., 2020a, 2020b; Bozkurt and Chaurasia, 2021; El-Ghandour et al., 2021; Clark et al., 2022; Hoz et al., 2021; Srinivasan et al., 2022; Suliman et al., 2022; Varela et al., 2022; Garg et al., 2022). Furthermore, we retrieved 57 published papers were produced through NC members collaborations (Suppl. Table 1). Thirteen studies were published through the “Neurosurgery platform” group (suppl. Table 2), 12 through the group named “Neurosurgery” (suppl. Table 3). We qualitatively analyzed the type of studies produced and the international collaborations. Results are summarized in Table 2.

**Table 2:** Quantitative and qualitative metrics of publications from major neurosurgical groups in Facebook.

Group name	Total papers	Basic research	General neurosurgery	Functional neurosurgery	Global Neurosurgery	Not classified	International collaboration
*Neurosurgery cocktail*	57	12	22	8	6	9	20 (35.1%)
*Neurosurgery*	12	4	3	4	0	1	7 (58.3%)
*Neurosurgery platform*	13	3	2	6	0	2	6 (46.2%)

## 4 Discussion

In this study, we analyzed activities/interactions within the largest neurosurgery groups available in social media. We attempted to do a quantitative and qualitative analysis to identify the lights and shadows of a groundbreaking phenomenon. The largest group (Neurosurgery Cocktail) was established in 2016, and now counts over 35,000 followers worldwide, with almost 30,000 on Facebook. Neurosurgery Cocktail is an independent group prominent on multiple social media platforms and can be accessed on any digital device, making it easy to utilize in daily practice. Other 3 groups with >10K followers are currently available in Facebook (Table 1). The amount of published material, especially the clinical cases presented to the community, is however inferior (i.e. 5 in Neurosurgery versus 173 cases in NC). A couple of recently published papers have quantitatively analyzed the impact of this group in different social media platform (Chaurasia et al., 2022; LE et al., 2022).

The success of neurosurgical communities in SM indicates a specific need for interaction among neurosurgeons, who seem to look for a larger and more international environment to share aspirations, projects, professional issues, and to seek collaborations and support. This constant contact with a large and receptive community is certainly psychologically rewarding for users that can instantly receive feedback on their enquiries. Specialty communities are consulted daily, suggesting that it is a part of the user routine during the workweek instead of being only utilized during free time, as it is expected of SM platforms, witnessing an impact on working activities. Both Facebook and Twitter demonstrated high engagement from followers (Chaurasia et al., 2022; LE et al., 2022), suggesting that the propensity of social media to be utilized as an educational and idea-sharing space is not limited solely to one specific platform. Focusing on the largest group (NC), all different areas of the world are represented (LE et al., 2022). The American, Eastern Mediterranean, European, and South-East Asian regions were equally represented within the group, with a smaller but still significant number of members from the South Pacific and African regions, demonstrating the value of SM for global neurosurgery. The immediately evident benefit of such a large community with different geopolitical provenience is the possibility of having a realistic perspective on how the neurosurgical activity is conjugated in environments with very different cultural and economic backgrounds. For instance, treatment modalities, equipments availability, end-of-life management, use of resources can be easily interpreted form the posted material and relative comments. This represents a unique opportunity to obtain a real-world perspective of global neurosurgery, free from conventional filters represented by the normal “peer-review” or top-down modalities. In fact, these filters tend to select activities with higher treatment standards and authoritative institutions, but substantially do not provide a realistic view of neurosurgical activities in different economic and political contexts. Above all, we believe that such a realistic picture may allow for the identification of a basic core of education, skills and technology that can constitute a sustainable neurosurgery model and, as such, applicable in areas of the world more economically disadvantaged. Among different topics posted daily, clinical cases-category carried the highest number of reactions. This means that this is the way to better access colleagues from all around the globe to engage and start interacting. Although, about 1/3 of interactions were not relevant in terms of contribution to a clinical debate, it is noteworthy that for each published clinical case, hundreds of colleagues were reached who were interested to provide feedback and advice. Arguably, this represents the main mission of global neurosurgery and we advocate an attention to opportunities offered by social media in general and by these specific communities in particular.

Other positive social media applications in medicine include the promotion of public health, education of patients and trainees, tools for research collaborations, professional networking, and practice development. Users ability to engage directly with authors, journals, and universities opens a door for collaboration between groups that otherwise would not be feasible and social media presence is associated with higher academic impact metrics for both neurosurgical departments and journals (Linzey et al., 2020). NC promoted 50 surveys on the Covid-19 pandemic to quantify its impact on daily practice, 15 of which have since been published (Chaurasia et al., 2022; Fontanella et al., 2020; Deora et al., 2020, 2022; Tandon et al., 2021; Robertson et al., 2020a, 2020b; Bozkurt and Chaurasia, 2021; El-Ghandour et al., 2021; Clark et al., 2022; Hoz et al., 2021; Srinivasan et al., 2022; Suliman et al., 2022; Varela et al., 2022; Garg et al., 2022). Furthermore, 57 published papers were produced through collaborations among members (Suppl. Table 1). Others were published through the “Neurosurgery” and “Neurosurgery platform” groups (Table 2; suppl. Table 2 and 3). This collaborative approach represents an unprecedented opportunity that until now has allowed neurosurgeons from 4 continents and >50 Countries to meet and cooperate.

On the other hand, SM interactions do not come without significant risks. Issues of copyright infringement, patient data breach, insufficient data reporting, and the potential to stir controversy between users should always be considered, and careful steps should be taken to avoid these instances whenever possible. For example, social media platforms could incorporate algorithms that attend the particular needs of the medical community to guiding patient information disclosure.

Patient data breaches were disclosed in 50% of clinical case posts. The privacy infringement through social media is particularly relevant, because the posting body has no control to amend previously posted content. Namely, once private data are submitted online, they are substantially irrevocable (Mata et al., 2016; Lifchez et al., 2012). The information can be potentially circulated to wide and possibly unintended audiences (Lagu and Greysen, 2011; Shore et al., 2011; Snyder, 2011). Even if the content has been de-identified, it could still potentially be traced back to specific patients if the content contains sufficiently unique identifiers such as a particular time period, institution, practitioner or limited geographic reach. Thus, irresponsible social media usage can cross professional boundaries, serve as a conduit for displaying unprofessional behavior, contribute to an irreversibly infamous online image, and subsequently lead to litigation, and legal consequences (Dmytriw et al., 2019).

The second critical aspect concerns the quality of posted material. Lack of sufficient clinical data and follow up were disclosed in up of 60% of posted material leading to the inability of the audience to give or receive any reliable and robust contribution to the clinical case discussion. This means the loss of a great opportunity for the community members to learn and for patients to benefit from an improved practice. Correct reporting of clinical, follow up and complications details are essential as well as is adherence to ethical standards. Most physicians are unlikely to post cases with complications or poor clinical outcomes. Instead, they are probably much more likely to post successful, complex or technically challenging procedures than routine surgeries. Therefore, social media posts may potentially mischaracterize practice standards to the audience. For example, in a study evaluating stroke thrombectomy cases posted on Twitter by physicians, posted cases had higher reperfusion scores and better outcomes than multiple randomized trials. None of the 115 cases posted on social media reported complications, post-procedure hemorrhages, or patient mortality (Dmytriw et al., 2019).

Also, social media serves as a potential advertising mechanism and may help establish an individual or entity's reputation in the community. Therefore, physicians or their associates who elect to post clinical cases on social media should be aware of the potential to mislead the general public regarding acceptable clinical practices, outcomes, and treatments.

### 4.1 The road ahead

Although relatively new, SM is playing an emerging, if not dominant, role as an information channel for continuous education, peer and patient engagement, benchmarking opinions, enhancing professional networking and communication, and as an increasingly important research tool (Atherton and Majeed, 2011; Chen et al., 2016). External factors such as the global pandemic have evidenced these changes, but substantially just accelerated the process. Indeed, almost all medical students and residents use social media, implying that in a few years, most practicing physicians will have had significant exposure (Bosslet et al., 2011).

The adoption of social media in medicine falls almost expectedly and stereotypically under the axiomatic assumption that new things are inherently flawed. Our study spotted and quantified some of these flaws and limitations, that are however intrinsic to the nature of social media and its bottom-up approach. Risks and flaws particularly concerned data breach, insufficient quality of case materials, and biased results reporting. There are possible actions now undertaken to correct these flaws, especially to provide a greater credibility and overall efficacy to the system. These include: the administrators of the platform now review posted material before publication; grids of basic information have been created for case reports, follow up data and complications report; attention during review is paid to respect of quality of published material and personal data protection.

Furthermore, in such large communities, flaws are automatically amended by the social interactions that quickly provide feedback on the post quality and their real value to the community. In other words, the interactive and collective nature of the media limits major flaws by itself. Such an opportunity is not available using conventional channels that are managed with a “top down” approach and without interaction and, as such, require strict and careful rules to preserve any scientific value. Although, initiatives to improve quality of posts are ongoing, we believe that the community “peer-revieweing” is the most powerful instrument to warrant veracity, value, and accountability of published material.

## 5 Conclusions

Social media have demonstrated the capability to disrupt social, political and economic barriers among peoples. The advantages to our discipline are hard to envision but the opportunity to boost international collaborations and to provide a realistic overview of neurosurgery practice in different areas of the world are probably the most evident. On the other hand, risks and pitfalls when applied to medical activities should be considered. There are currently relevant issues with privacy of personal information. It was also evident that in a large number of cases, clinical information was insufficiently provided, so that no significant benefits were obtained from many posted materials. Similar to the almost ubiquitous disclaimer found appended to most published conclusions, resources obtained from those platforms should be taken with caution. However, the diffusion of the SM community model is unavoidable and the benefits greatly overcome limitations. Adjustments are necessary to help submitters to provide useful contributions to achieve the support from the community and avoid medico-legal issues. Apart from this, the “power of the collective” is a self-adjusting modality for providing a strong and equitable opinion and scientific contribution that has already changed our education and perception of neurosurgery.

## Declaration of competing interest

The authors declare that they have no known competing financial interests or personal relationships that could have appeared to influence the work reported in this paper.
