# Supplementary material for: Social Media for Global Neurosurgery. Benefits and limitations of a groundbreaking approach to communication and education
Source: Brain Spine. 2023 Mar 11;3:101728. doi: 10.1016/j.bas.2023.101728 (PMC10293234; doi:10.1016/j.bas.2023.101728)
Supplement: Multimedia component 1 [file mmc1.docx]

| **N°** | **Year** | **Title** | **Journal** | **ROI** | **International collaboration** | **DOI** |
| --- | --- | --- | --- | --- | --- | --- |
| 1 | 2022 | Nociceptor neurons direct goblet cells via a CGRP-RAMP1 axis to drive mucus production and gut barrier protection | Cell | Harvard – Chicago (USA) | None | - [10.1016/j.cell.2022.09.024](https://doi.org/10.1016/j.cell.2022.09.024) |
| 2 | 2022 | Initial experience with the contour device in the treatment of ruptured intracranial wide-necked bifurcation aneurysms: a single-operator multicenter study | Neurology India | Mumbai (India) | None | - [10.4103/0028-3886.359281](https://doi.org/10.4103/0028-3886.359281) |
| 3 | 2022 | Evolution in Cerebrovascular Bypass: Conceptual Framework, Technical Nuances, and Initial Clinical Experience with Fourth-Generation Bypass | Neurosurgery clinics of North America | Pittsburgh (USA) | None | - [10.1016/j.nec.2022.06.004](https://doi.org/10.1016/j.nec.2022.06.004) |
| 4 | 2022 | Structure-based discovery of non-opioid analgesics acting through the a2A-adrenergic receptor | Science | US, China, Germany, Ukraine, Canada, Latvia | Yes | - [10.1126/science.abn7065](https://doi.org/10.1126/science.abn7065) |
| 5 | 2022 | Intracranial direct electrical mapping reveals the functional architecture of the human basal ganglia | Communication biology | China | None | - [10.1038/s42003-022-04084-3](https://doi.org/10.1038/s42003-022-04084-3) |
| 6 | 2022 | Decompression and fusion surgery for osteoporotic vertebral fractures: WFNS spine committee recommendations | Journal of Neurosurgical Sciences | Turkey - Pakistan | Yes | - [10.23736/S0390-5616.22.05640-5](https://doi.org/10.23736/s0390-5616.22.05640-5) |
| 7 | NA | NA | NA | NA | NA | NA |
| 8 | 2022 | Predictors of surgical site infection in glioblastoma patients undergoing craniotomy for tumor resection | Journal of Neurosurgery | Not available | Not available | - [10.3171/2022.8.JNS212799](https://doi.org/10.3171/2022.8.jns212799) |
| 9 | 2022 | Narcolepsy-A Neuropathological Obscure Sleep Disorder: A Narrative Review of Current Literature | Brain Sciences | USA, Nepal, Italy, Austria | Yes | - [10.3390/brainsci12111473](https://doi.org/10.3390/brainsci12111473) |
| 10 | NA | NA | NA | NA | NA | NA |
| 11 | 2022 | Initial experience with the contour device in the treatment of ruptured intracranial wide-necked bifurcation aneurysms: a single-operator multicenter study | Neurology India | Mumbai (India) | None | - [10.4103/0028-3886.359281](https://doi.org/10.4103/0028-3886.359281) |
| 12 | 2022 | Adult-born dentate granule cells promote hippocampal population sparsity | Nature neuroscience | Oxford (UK) | None | - [10.1038/s41593-022-01176-5](https://doi.org/10.1038/s41593-022-01176-5) |
| 13 | 2022 | Cranio-Orbital Pretemporal Approach With Extradural Anterior Clinoidectomy and Optic Nerve Release for Microsurgical Resection of Large Tuberculum Sellae Meningioma-Reversal of Preoperative Bilateral Blindness: 2-Dimensional Operative Video | Operative Neurosurgery | USA, Croatia | Yes | - [10.1227/ons.0000000000000519](https://doi.org/10.1227/ons.0000000000000519) |
| 14 | 2021 | Validation of the Clavien-Dindo grading system of complications for microsurgical treatment of unruptured intracranial aneurysms | Neurosurgical focus | Switzerland | None | - [10.3171/2021.8.FOCUS20892](https://doi.org/10.3171/2021.8.focus20892) |
| 15 | 2022 | Cisternal, Falciform, and Optic Canal Decompression Influencing Optic Nerve Biomechanics: A Microsurgical Anatomic Study | Operative Neurosurgery | USA | None | - [10.1227/ons.0000000000000472](https://doi.org/10.1227/ons.0000000000000472) |
| 16 | NA | NA | NA | NA | NA | NA |
| 17 | 2022 | kHz-frequency electrical stimulation selectively activates small, unmyelinated vagus afferents | Brain stimulation | USA - Australia | Yes | - [10.1016/j.brs.2022.09.015](https://doi.org/10.1016/j.brs.2022.09.015) |
| 18 | NA | NA | NA | NA | NA | NA |
| 19 | 2022 | Standing e-scooters, a new self-harming tool in the motor vehicle accidents TBI chapter: do we really deserve it? | Journal of Neurosurgical Sciences | Italy | None | - [10.23736/S0390-5616.22.05676-4](https://doi.org/10.23736/s0390-5616.22.05676-4) |
| 20 | 2022 | The Role of Social Media on the Research Productivity of Neurosurgeons During the COVID-19 Pandemic | World Neurosurgery | USA, Turkey, Nepal, Italy | Yes | - [10.1016/j.wneu.2022.09.051](https://doi.org/10.1016/j.wneu.2022.09.051) |
| 21 | 2021 | Elderly traumatic central cord syndrome in the USA: a review of management and outcomes | Journal of Neurosurgical Sciences | USA | None | - [10.23736/S0390-5616.21.05078-5](https://doi.org/10.23736/s0390-5616.21.05078-5) |
| 22 | NA | NA | NA | NA | NA | NA |
| 23 | 2022 | Effect of Spinal Cord Burst Stimulation vs Placebo Stimulation on Disability in Patients With Chronic Radicular Pain After Lumbar Spine Surgery: A Randomized Clinical Trial | JAMA | Norway, Sweden | Yes | - [10.1001/jama.2022.18231](https://doi.org/10.1001/jama.2022.18231) |
| 24 | 2016 | Delivery of ziconotide to cerebrospinal fluid via intranasal pathway for the treatment of chronic pain | Journal of controlled release | USA, India | Yes | - [10.1016/j.jconrel.2015.12.044](https://doi.org/10.1016/j.jconrel.2015.12.044) |
| 25 | NA | NA | NA | NA | NA | NA |
| 26 | 2022 | The medico-legal implications of intraoperative neurophysiological monitoring, in compliance with good clinical-assistance practices, according to the current Italian legal system | Journal of Neurosurgical Sciences | Italy | None | - [10.23736/S0390-5616.22.05734-4](https://doi.org/10.23736/s0390-5616.22.05734-4) |
| 27 | 2022 | Prospective Randomized Control Trial to Compare the Role of Injection Cerebrolysin for 10 Days Duration Against Placebo in Operated Cases of Degenerative Cervical Myelopathy | Spine | India | None | - [10.1097/BRS.0000000000004542](https://doi.org/10.1097/brs.0000000000004542) |
| 28 | 2020 | The seven miracles of neurosurgery | Journal of Neurosurgical sciences | Greece | None | - [10.23736/S0390-5616.19.04855-0](https://doi.org/10.23736/s0390-5616.19.04855-0) |
| 29 | 2022 | Awareness regarding the use of topical vancomycin in cranial and spinal surgeries in developing countries | Journal of Neurosurgical sciences | India, USA, Nepal | Yes | - [10.23736/S0390-5616.22.05840-4](https://doi.org/10.23736/s0390-5616.22.05840-4) |
| 30 | 2022 | Radiotherapy or systemic therapy versus combined therapy in patients with brain metastases: a propensity-score matched study | Journal of Neuro-Oncology | Japan | None | - [10.1007/s11060-022-04132-2](https://doi.org/10.1007/s11060-022-04132-2) |
| 31 | 2022 | Clinical utilization of fast-acting sub-perception therapy (FAST) in SCS-implanted patients for treatment of mixed pain | Interventional pain medicine | Germany, USA | Yes | <https://doi.org/10.1016/j.inpm.2022.100165> |
| 32 | 2022 | Acquired Chiari type I malformation: a late and misunderstood supratentorial over-drainage complication | Child’s nervous system | Italy | None | - [10.1007/s00381-022-05775-2](https://doi.org/10.1007/s00381-022-05775-2) |
| 33 | 2021 | Increased proliferation is associated with CNS invasion in meningiomas | Journal of Neuro-Oncology | Germany | None | - [10.1007/s11060-021-03892-7](https://doi.org/10.1007/s11060-021-03892-7) |
| 34 | 2022 | Letter to the Editor Regarding "Management of Hydrocephalus with Ventriculoperitoneal Shunts: Review of 109 Cases of Children" | World Neurosurgery | Russia, Nepal, Uganda | Yes | - [10.1016/j.wneu.2022.04.085](https://doi.org/10.1016/j.wneu.2022.04.085) |
| 35 | 2022 | Decompressive Craniectomy Practice following Traumatic Brain Injury in Comparison with Randomized Trials: Harmonized, Multi-Center Cohort Studies in Europe, the United Kingdom, and Australia | Journal of Neurotrauma | Australia, The Netherlands, UK, Belgium | Yes | - [10.1089/neu.2021.0312](https://doi.org/10.1089/neu.2021.0312) |
| 36 | 2021 | Increased proliferation is associated with CNS invasion in meningiomas | Journal of Neuro-Oncology | Germany | None | - [10.1007/s11060-021-03892-7](https://doi.org/10.1007/s11060-021-03892-7) |
| 37 | 2022 | Unilateral facet cyst at the atlantoaxial joint leading to cervical myelopathy: A case report and review of literature | Surgical Neurology International | India, Mexico | Yes | - [10.25259/SNI_900_2022](https://doi.org/10.25259/sni_900_2022) |
| 38 | 2022 | Role of interhemispheric connectivity in recovery from postoperative supplementary motor area syndrome in glioma patients | Journal of Neurosurgery | Germany | None | - [10.3171/2022.10.JNS221303](https://doi.org/10.3171/2022.10.jns221303) |
| 39 | 2022 | The Effect of Losartan on Neuroinflammation as Well as on Endothelin-1- and Serotonin-Induced Vasoconstriction in a Double-Haemorrhage Rat Model | Journal of Clinical Medicine | Germany, Switzerland | Yes | - [10.3390/jcm11247367](https://doi.org/10.3390/jcm11247367) |
| 40 | 2022 | Olfactory Groove Meningiomas: Comprehensive assessment between the different microsurgical transcranial approaches and the Endoscopic Endonasal Approaches, systematic review and metanalysis on behalf of the EANS skull base section | Brain and Spine | Spain, Venezuela, Chile, France, Germany, Switzerland, Italy, UK, The Netherlands, USA, Belgium | Yes | - [10.1016/j.bas.2022.101661](https://doi.org/10.1016/j.bas.2022.101661) |
| 41 | 2022 | Understanding anterior communicating artery aneurysms: A bibliometric analysis of top 100 most cited articles | Journal of cerebrovascular and endovascular neurosurgery | Nepal, India | Yes | - [10.7461/jcen.2022.E2022.01.001](https://doi.org/10.7461/jcen.2022.e2022.01.001) |
| 42 | 2022 | Minimally invasive percutaneous anterior odontoid screw fixation: institutional experience with a simple and effective technique | Journal of Neurosurgical Sciences | Italy | None | - [10.23736/S0390-5616.20.04886-9](https://doi.org/10.23736/s0390-5616.20.04886-9) |
| 43 | 2022 | Variations in the neurosurgical management of depressed skull fractures in adults: An international cross-sectional survey | Journal of Neurological Surgery | UK, Northern Ireland, Nepal | Yes | - [10.1055/a-1994-9330](https://doi.org/10.1055/a-1994-9330) |
| 44 | 2017 | Crossed Aphasia in a Patient with Anaplastic Astrocytoma of the Non-Dominant Hemisphere | Journal of Radiology Case Reports | USA | None | - [10.3941/jrcr.v11i9.3154](https://doi.org/10.3941/jrcr.v11i9.3154) |
| 45 | 2020 | Basal ganglia role in learning rewarded actions and executing previously learned choices: Healthy and diseased states | PloS one | USA | None | - [10.1371/journal.pone.0228081](https://doi.org/10.1371/journal.pone.0228081) |
| 46 | 2022 | Neutrophil-lymphocyte ratio as a predictor of outcome following traumatic brain injury: Systematic review and meta-analysis | Journal of Neurosciences in rural practice | India, Colombia, Spain, USA | Yes | - [10.25259/JNRP-2022-4-21](https://doi.org/10.25259/jnrp-2022-4-21) |
| 47 | 2020 | A blood-brain barrier overview on structure, function, impairment, and biomarkers of integrity | Fluid and barriers of the CNS | USA | None | - [10.1186/s12987-020-00230-3](https://doi.org/10.1186/s12987-020-00230-3) |
| 48 | 2022 | Prognostic factors affecting outcome of multifocal or multicentric glioblastoma: A scoping review | Journal of Neurosciences in rural practice | India | None | [10.25259/JNRP_41_2022](https://ruralneuropractice.com/prognostic-factors-affecting-outcome-of-multifocal-or-multicentric-glioblastoma-a-scoping-review/) |
| 49 | 2022 | Letter to the Editor Regarding "Sellar Xanthogranuloma: A Quest Based on 9 Cases Assessed with an Anterior Pituitary Provocation Test" | World Neurosurgery | India | None | - [10.1016/j.wneu.2022.09.065](https://doi.org/10.1016/j.wneu.2022.09.065) |
| 50 | 2022 | Intracranial pressure monitoring in craniosynostosis | Journal of Neurosciences in rural practice | India | None | [http://dx.doi.org/10.25259/JNRP-2022-6-41](https://www.researchgate.net/publication/365594346_Intracranial_pressure_monitoring_in_craniosynostosis) |
| 51 | 2022 | Letter: Global Neurosurgery: The Pakistani Perspective | Neurosurgery | Pakistan, USA, Nepal | Yes | - [10.1227/neu.0000000000002265](https://doi.org/10.1227/neu.0000000000002265) |
| 52 | 2022 | The EANS Young Neurosurgeons Committee's vision of the future of European Neurosurgery | Journal of Neurosurgical Sciences | Italy, Romania, Sweden, Denmark, Israel, Portugal, UK, Austria, Russia, Serbia, Germany, Bulgaria, Switzerland, Spain | Yes | - [10.23736/S0390-5616.22.05802-7](https://doi.org/10.23736/s0390-5616.22.05802-7) |
| 53 | 2018 | Central Pain Syndrome: Etiological Perspectives from the 3D Default Space Model of Consciousness | World Journal of Neuroscience | USA | None | [https://doi.org/10.4236/wjns.2018.82022](https://www.scirp.org/journal/paperinformation.aspx?paperid=84489) |
| 54 | 2022 | Letter to the Editor Regarding "Evacuation of Chronic Subdural Hematoma: Case Series and Literature Review" | World Neurosurgery | Nepal | None | - [10.1016/j.wneu.2022.09.107](https://doi.org/10.1016/j.wneu.2022.09.107) |
| 55 | 2022 | Cerebrospinal fluid dynamics along the optic nerve | Frontiers in Neurology | China | None | - [10.3389/fneur.2022.931523](https://doi.org/10.3389/fneur.2022.931523) |
| 56 | NA | NA | NA | NA | NA | NA |
| 57 | 2022 | SPECT/CT imaging for diagnosis and management of failed cervical spine surgery syndrome | Interdisciplinary Neurosurgery | USA | None | <https://doi.org/10.1016/j.inat.2022.101699> |
