# Supplementary material for: Social Media for Global Neurosurgery. Benefits and limitations of a groundbreaking approach to communication and education
Source: Brain Spine. 2023 Mar 11;3:101728. doi: 10.1016/j.bas.2023.101728 (PMC10293234; doi:10.1016/j.bas.2023.101728)
Supplement: Multimedia component 2 [file mmc2.docx]

| **N°** | **Year** | **Title** | **Journal** | **ROI** | **International collaboration** | **DOI** |
| --- | --- | --- | --- | --- | --- | --- |
| 1 | 2022 | Nociceptor neurons direct goblet cells via a CGRP-RAMP1 axis to drive mucus production and gut barrier protection | Cell | Harvard – Chicago (USA) | None | - [10.1016/j.cell.2022.09.024](https://doi.org/10.1016/j.cell.2022.09.024) |
| 2 | 2022 | Intracranial direct electrical mapping reveals the functional architecture of the human basal ganglia | Communication Biology | China | None | - [10.1038/s42003-022-04084-3](https://doi.org/10.1038/s42003-022-04084-3) |
| 3 | 2022 | Structure-based discovery of non-opioid analgesics acting through the a2A-adrenergic receptor | Science | US, China, Germany, Ukraine, Canada, Latvia | Yes | - [10.1126/science.abn7065](https://doi.org/10.1126/science.abn7065) |
| 4 | 2022 | Implementation of sacral neuromodulation for urinary indications. A Danish prospective study during the initial 15 months of a new service in a tertiary referral hospital | Scandinavian Journal of Urology | Denmark | None | - [10.1080/21681805.2022.2120066](https://doi.org/10.1080/21681805.2022.2120066) |
| 5 | NA | NA | NA | NA | NA | NA |
| 6 | 2022 | Three-Year Durability of Restorative Neurostimulation Effectiveness in Patients With Chronic Low Back Pain and Multifidus Muscle Dysfunction | Neuromodulation | USA, Australia, UK, Belgium, The Netherlands, Ireland | Yes | - [10.1016/j.neurom.2022.08.457](https://doi.org/10.1016/j.neurom.2022.08.457) |
| 7 | 2022 | kHz-frequency electrical stimulation selectively activates small, unmyelinated vagus afferents | Brain stimulation | USA, Australia | Yes | - [10.1016/j.brs.2022.09.015](https://doi.org/10.1016/j.brs.2022.09.015) |
| 8 | 2016 | Delivery of ziconotide to cerebrospinal fluid via intranasal pathway for the treatment of chronic pain | Journal of controlled release | USA, India | Yes | - [10.1016/j.jconrel.2015.12.044](https://doi.org/10.1016/j.jconrel.2015.12.044) |
| 9 | 2022 | Effect of Spinal Cord Burst Stimulation vs Placebo Stimulation on Disability in Patients With Chronic Radicular Pain After Lumbar Spine Surgery: A Randomized Clinical Trial | JAMA | Norway, Sweden | Yes | - [10.1001/jama.2022.18231](https://doi.org/10.1001/jama.2022.18231) |
| 10 | 2018 | Spinal Cord Infarction Because of Spontaneous Vertebral Artery Dissection | Stroke | USA | None | - [10.1161/STROKEAHA.118.022333](https://doi.org/10.1161/strokeaha.118.022333) |
| 11 | 2022 | Clinical utilization of fast-acting sub-perception therapy (FAST) in SCS-implanted patients for treatment of mixed pain | Interventional Pain Medicine | Germany, USA | Yes | <https://doi.org/10.1016/j.inpm.2022.100165> |
| 12 | 2022 | The American Society of Pain and Neuroscience (ASPN) Evidence-Based Clinical Guideline of Interventional Treatments for Low Back Pain | Journal of pain research | USA | None | - [10.2147/JPR.S386879](https://doi.org/10.2147/jpr.s386879) |
| 13 | NA | NA | NA | NA | NA | NA |
