# Supplementary material for: Social Media for Global Neurosurgery. Benefits and limitations of a groundbreaking approach to communication and education
Source: Brain Spine. 2023 Mar 11;3:101728. doi: 10.1016/j.bas.2023.101728 (PMC10293234; doi:10.1016/j.bas.2023.101728)
Supplement: Multimedia component 3 [file mmc3.docx]

| **N°** | **Year** | **Title** | **Journal** | **ROI** | **International collaboration** | **DOI** |
| --- | --- | --- | --- | --- | --- | --- |
| 1 | 2022 | Structure-based discovery of non-opioid analgesics acting through the a2A-adrenergic receptor | Science | US, China, Germany, Ukraine, Canada, Latvia | Yes | - [10.1126/science.abn7065](https://doi.org/10.1126/science.abn7065) |
| 2 | 2022 | Evolution in Cerebrovascular Bypass: Conceptual Framework, Technical Nuances, and Initial Clinical Experience with Fourth-Generation Bypass | Neurosurgery clinics of North America | Pittsburgh (USA) | None | - [10.1016/j.nec.2022.06.004](https://doi.org/10.1016/j.nec.2022.06.004) |
| 3 | 2022 | Nociceptor neurons direct goblet cells via a CGRP-RAMP1 axis to drive mucus production and gut barrier protection | Cell | Harvard – Chicago (USA) | None | - [10.1016/j.cell.2022.09.024](https://doi.org/10.1016/j.cell.2022.09.024) |
| 4 | 2022 | Intracranial direct electrical mapping reveals the functional architecture of the human basal ganglia | Communication Biology | China | None | - [10.1038/s42003-022-04084-3](https://doi.org/10.1038/s42003-022-04084-3) |
| 5 | 2022 | Decompression and fusion surgery for osteoporotic vertebral fractures: WFNS spine committee recommendations | Journal of Neurosurgical Sciences | Turkey - Pakistan | Yes | - [10.23736/S0390-5616.22.05640-5](https://doi.org/10.23736/s0390-5616.22.05640-5) |
| 6 | NA | NA | NA | NA | NA | NA |
| 7 | 2022 | kHz-frequency electrical stimulation selectively activates small, unmyelinated vagus afferents | Brain Stimulation | USA, Australia | Yes | - [10.1016/j.brs.2022.09.015](https://doi.org/10.1016/j.brs.2022.09.015) |
| 8 | 2022 | Self-sustainable intermittent deep brain stimulator | Cell reports physical science | USA, Egypt, Switzerland, Ireland | Yes | <https://doi.org/10.1016/j.xcrp.2022.101099> |
| 9 | 2016 | Delivery of ziconotide to cerebrospinal fluid via intranasal pathway for the treatment of chronic pain | Journal of controlled release | USA, India | Yes | - [10.1016/j.jconrel.2015.12.044](https://doi.org/10.1016/j.jconrel.2015.12.044) |
| 10 | 2022 | Clinical utilization of fast-acting sub-perception therapy (FAST) in SCS-implanted patients for treatment of mixed pain | Interventional pain medicine | Germany, USA | Yes | <https://doi.org/10.1016/j.inpm.2022.100165> |
| 11 | 2022 | The American Society of Pain and Neuroscience (ASPN) Evidence-Based Clinical Guideline of Interventional Treatments for Low Back Pain | Journal of pain research | USA | None | - [10.2147/JPR.S386879](https://doi.org/10.2147/jpr.s386879) |
| 12 | 2022 | The Effect of Losartan on Neuroinflammation as Well as on Endothelin-1- and Serotonin-Induced Vasoconstriction in a Double-Haemorrhage Rat Model | Journal of Clinical Medicine | Germany, Switzerland | Yes | - [10.3390/jcm11247367](https://doi.org/10.3390/jcm11247367) |
